# 本科生创新训练实验：动物考古质谱分析用于古生物遗存碎片的种属鉴定

**DOI:** 10.3724/SP.J.1123.2025.03003

**Published:** 2025-11-08

**Authors:** Yang XU, Liyan JIANG, Shasha YANG

**Affiliations:** 吉林大学生命科学学院，吉林 长春 130012; College of Life Sciences，Jilin University，Changchun 130012，China

**Keywords:** 创新训练实验, 动物考古质谱分析, 古生物遗存碎片, 种属鉴定, 基质辅助激光解吸电离飞行时间质谱, innovation training experiment, zooarchaeology by mass spectrometry （ZooMS）, paleontology remnants, species identification, matrix-assisted laser desorption/ionization time-of-flight mass spectrometry （MALDI-TOF MS）

## Abstract

在学科深度交叉融合，赋能复合型人才培养新模式的教育背景下，本创新训练实验着眼于分子考古学的热点交叉学科，针对呈现碎片状态、不具备形态学鉴定特征、无法进行准确种属鉴定的古生物遗存，基于基质辅助激光解吸电离飞行时间质谱，利用胶原蛋白肽质量指纹图谱开发动物考古质谱分析（ZooMS）方法，并成功应用于晚更新世古生物遗存碎片样本的种属鉴定研究。ZooMS具有操作便捷、鉴定精度高、适用范围广、测试成本低、高通量检测、严控污染的显著优势，在古生物遗存碎片的种属鉴定及“潜信息”挖掘与重建研究中具有重要价值。通过该创新训练实验的开展，加强了文理学科之间的相互渗透和深度融合，激发了学生的科学思维，提高了学生的科研素质，为复合型创新人才的培养奠定了坚实基础。

## Abstract (en)

Against the educational backdrop of deep interdisciplinary integration and empowering new models for cultivating composite talents， this innovative training experiment focuses on the hot interdisciplinary field of molecular archaeology， targeting the fragmented state of paleontological remains that do not have morphological identification characteristics and cannot be accurately identified for species. We developed a zooarchaeology by mass spectrometry （ZooMS） method based on matrix-assisted laser desorption/ionization time-of-flight mass spectrometry （MALDI-TOF MS）， utilizing collagen peptide mass fingerprinting （PMF）. This approach was successfully applied to species identification of late pleistocene fragmented paleontological samples. ZooMS offers significant advantages including operational simplicity， high identification accuracy， broad applicability， low testing costs， high-throughput analysis and stringent contamination control， making it highly valuable for species identification and the extraction of “hidden information” from fragmented paleontological remains. Through this innovation training experiment， we enhanced interdisciplinary collaboration between humanities and sciences， stimulated students’ scientific thinking， and improved their research competencies， laying a solid foundation for cultivating interdisciplinary and innovative talents.

习近平总书记在中央人才工作会议上强调，“双一流”大学要发挥培养基础研究人才主力军作用，全方位谋划培养高水平复合型人才^［1，2］^。目前，世界一流大学和一流学科建设加速推进，各高校深耕多学科间的交叉融合，努力将人才培养模式向多学科交叉的大类培养模式转变，积极探索具有创新精神和综合实践能力的复合型人才培养策略^［1］^。创新训练课程作为热点教学模式，不仅能够巩固学生的基础知识和实验技能，而且能通过解决实际问题，提升学生的综合应用能力，因此逐渐受到各个高校的关注^［3］^。本实验着眼于分子考古学的热点交叉学科，将分子生物学技术与生物考古学交叉融合整合入本科实验教学，以培养能够将自然科学技术手段和考古探索有机结合起来的高素质复合型人才。

动物考古质谱分析（zooarchaeology by mass spectrometry，ZooMS）技术是基质辅助激光解吸电离飞行时间质谱（MALDI-TOF MS）的肽质量指纹图谱检测手段在动物考古领域的拓展应用，即通过检测动物骨骼或牙齿中残留的胶原蛋白实现对未知样本的种属鉴定^［4-6］^。胶原蛋白是古代动物样本中除无机物外保存情况最为完整的大分子物质，也是动物体内含量最丰富的结构蛋白^［7，8］^，其包含多种类型，最常见的是Ⅰ型胶原蛋白，由3条α肽链（两条α1链和一条α2链）通过互相缠绕形成3股螺旋结构，然后聚集成微纤维，再聚集形成原纤维，最终束在一起形成胶原纤维^［9，10］^，这种独特的分子结构在自然环境中具有较强的稳定性，因此能够在埋藏百万年的生物样本中留存下来。不同物种的胶原蛋白在氨基酸组成上具有特异性，这种特异性如同“指纹”一般。ZooMS技术通过将肽质量指纹图谱中的特征峰出现的位置（*m/z*）与特定的胶原蛋白序列进行关联，从而识别出不同种属之间胶原蛋白序列差异，最终实现种属的鉴定^［8］^，其分析原理示意如图1。

**图1:**
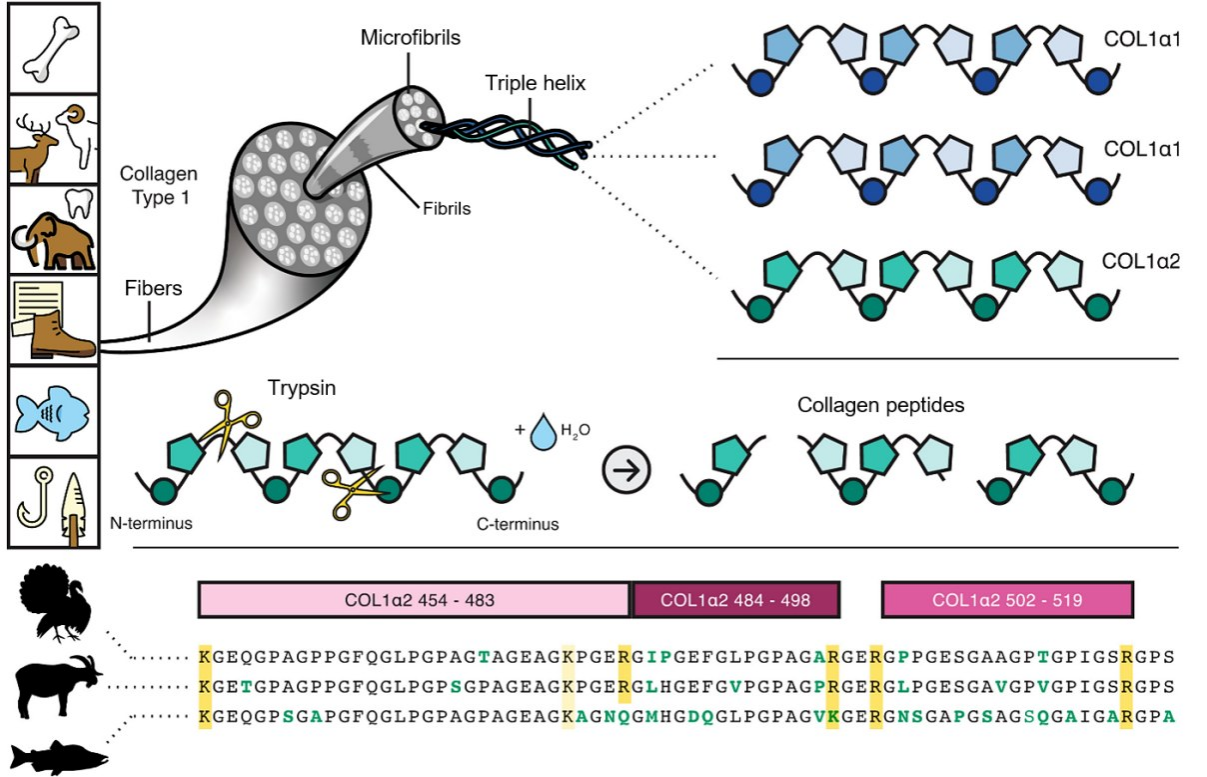
ZooMS分析的原理示意图^［5］^

ZooMS技术进行数据分析的关键是将实验谱图与哺乳动物胶原蛋白数据库进行比对（https：//docs.google.com/spreadsheets/d/1ipm9fFFyha8 IEzRO2F5zVXIk0ldwYiWgX5pGqETzBco/edit？gid=1005946405#gid=1005946405）。该数据库中有9条特征肽段（A~G， P1， P2），参考Brown等^［9］^2021年提出的标准化命名法，9条肽段对应的区域分别为：A=COL1α2 978~990， B=COL1α2 484~498， C=COL1α2 502~519， D=COL1α2 793~816， E=COL1α2 454~483， F=COL1α1 586~618， G=COL1α2 757~789， P1=COL1α1 508~519， P2=COL1α2 292~309。在谱图中检测到上述9条特征肽段中的3条或3条以上，则认为鉴定结果可信^［6］^。

古生物遗存年代久远，碎片化严重，有效生物信息含量低甚至可能消失殆尽，无法采用更为成熟的古DNA分析手段或传统的形态学研究进行种属鉴定。因此，我们结合古生物遗存样本特点，将ZooMS方案进行优化，拓展应用于晚更新世古生物遗存碎片的种属鉴定。我们的实验结果表明，该方法适用于晚更新世古生物遗存碎片的鉴定，为古生物遗存的种属鉴定提供了新的策略。

## 1 实验部分

### 1.1 仪器、试剂与材料

5800基质辅助激光解吸电离飞行时间质谱仪（AB SCIEX公司，美国），384 MALDI靶板（AB SCIEX公司，美国），DLX-TPC110电子天平（德力西电气公司，中国），DHP-9012恒温培养箱（一恒上科仪公司，中国），SW-VD-650超净工作台（上海艾析公司，中国），L-100XP 高速离心机（Beckman公司，美国），1010F-K 喷砂机（大连创远机械设备公司，中国）。

次氯酸钠、无水乙醇和盐酸均为分析级，购于广东光华科技股份有限公司；碳酸氢铵溶液（ammonium bicarbonate buffer，AmBic，0.05 mol/L）、乙腈溶液、三氟乙酸溶液和胰蛋白酶溶液均为分析级，*α*-氰基-4-羟基肉桂酸溶液（CHCA）和质谱校准标准品均为色谱级，均购于德国Thermo公司。

### 1.2 质谱分析条件

待检测肽段的浓度为0.5 mg/mL，点样量0.5 µL，与CHCA基质（10 mg/mL）等比例混合，点于384靶板上，设空白样本作为阴性对照，每个样本重复点样3次。采用反射正离子检测模式，相对分子质量检测范围为800~3 500，激光波长349 nm，激光强度设定范围为3 500~5 000。

### 1.3 实验步骤

#### 1.3.1 样品选取及采集

研究晚更新世的动物群可以揭示早期人类与动物之间的关系，对于现代生态系统的管理和保护具有借鉴意义^［11］^。依托于吉林大学分子考古实验室提供的晚更新世时期形态上无法识别的古生物遗存碎片样本，指导学生对已储备的古生物遗存碎片样本逐一进行登记及编号。选取已通过蛋白质组学鉴定为骆驼属的3个样本（Camel-1、Camel-2和Camel-3 ）用于ZooMS技术方法的建立（图2）。

**图2:**
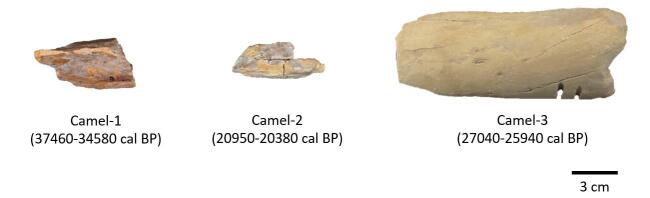
用于方法建立的骆驼属样本照片及测年数据

#### 1.3.2 样品预处理

为确保古生物研究数据的真实性和质量，严格的污染控制是古生物实验研究中的关键环节^［12］^。研究在符合ISO 5级洁净标准的古DNA实验室内进行，所有操作人员均穿戴医用防护服、医用外科口罩及双层无菌手套。实验前，所有试剂外表面均经无水乙醇及次氯酸钠（分析纯，4%~6%）系统擦拭消毒。实验流程如下：采用喷砂机对样本拟取样部位进行表面打磨以彻底去除外源污染，使用切割机切取10~20 mg样本置于1.5 mL EP管中^［6，13］^。每个样本处理完成后，依次以次氯酸钠溶液擦拭刀片切割部位并对切片进行喷砂处理，确保消除样本间交叉污染风险^［6］^。目前广泛应用的蛋白质提取方法大多采用有损提取^［14］^，我们在现有研究的基础上，针对古生物样本的特点对方法进行了双重优化，一方面在保证数据质量的前提下将样本需求量降至最低，另一方面增设了系列防污染控制步骤。

#### 1.3.3 胶原蛋白提取方法优化

胶原蛋白的稳定结构使其成为ZooMS技术在化石样本应用中的理想检测对象。由于古代样品会在埋藏过程中发生生物矿化现象，其内部具有较高含量的钙盐，因此在提取胶原蛋白前需进行脱矿处理。整体实验流程包括以下几个步骤，（1）脱矿：在放置实验样品的1.5 mL EP管中加入500 µL HCl溶液，室温静置2~5天；（2）提取：利用酸不溶性提取方法对样本中的蛋白进行提取，将样本于20 000 rcf 离心1 min，收集上清液冻存备份^［6］^；沉淀用AmBic 洗涤3次至中性，随后加入100 µL AmBic，置于65 ℃下孵育1 h使胶原凝胶化^［15］^；（3）酶解：孵育结束后离心1 min，将上清液转移至新的离心管中加入胰蛋白酶溶液，置于37 ℃恒温箱中酶解12~18 h后，离心1 min，并加入5%的三氟乙酸终止酶解^［6］^；（4）肽段回收：通过C18固相萃取柱纯化酶解肽段，最终获得所需检测的胶原蛋白肽段（图3）。

**图3:**
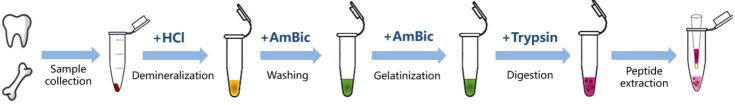
古生物遗存中胶原蛋白的提取

## 2 结果与讨论

### 2.1 质谱检测方法及检测模式的建立

采用1.3节中优化的方法对骆驼样本的胶原蛋白进行提取后，将纯化的肽段与基质混合，点于靶板，室温自然干燥，用于质谱测试^［16-18］^。学生通过反复试验，调节检测参数，确定最优的检测条件如下：待测肽段质量浓度为0.5 mg/mL，与CHCA基质（10 mg/mL）等体积混合，室温自然结晶后，点于靶板上，点样量为0.5 µL。以空白样本作为阴性对照，每个样本重复点样3次。在该检测条件下，3个测试样本均获得了较好的肽质量指纹图谱（图4）。

**图4:**
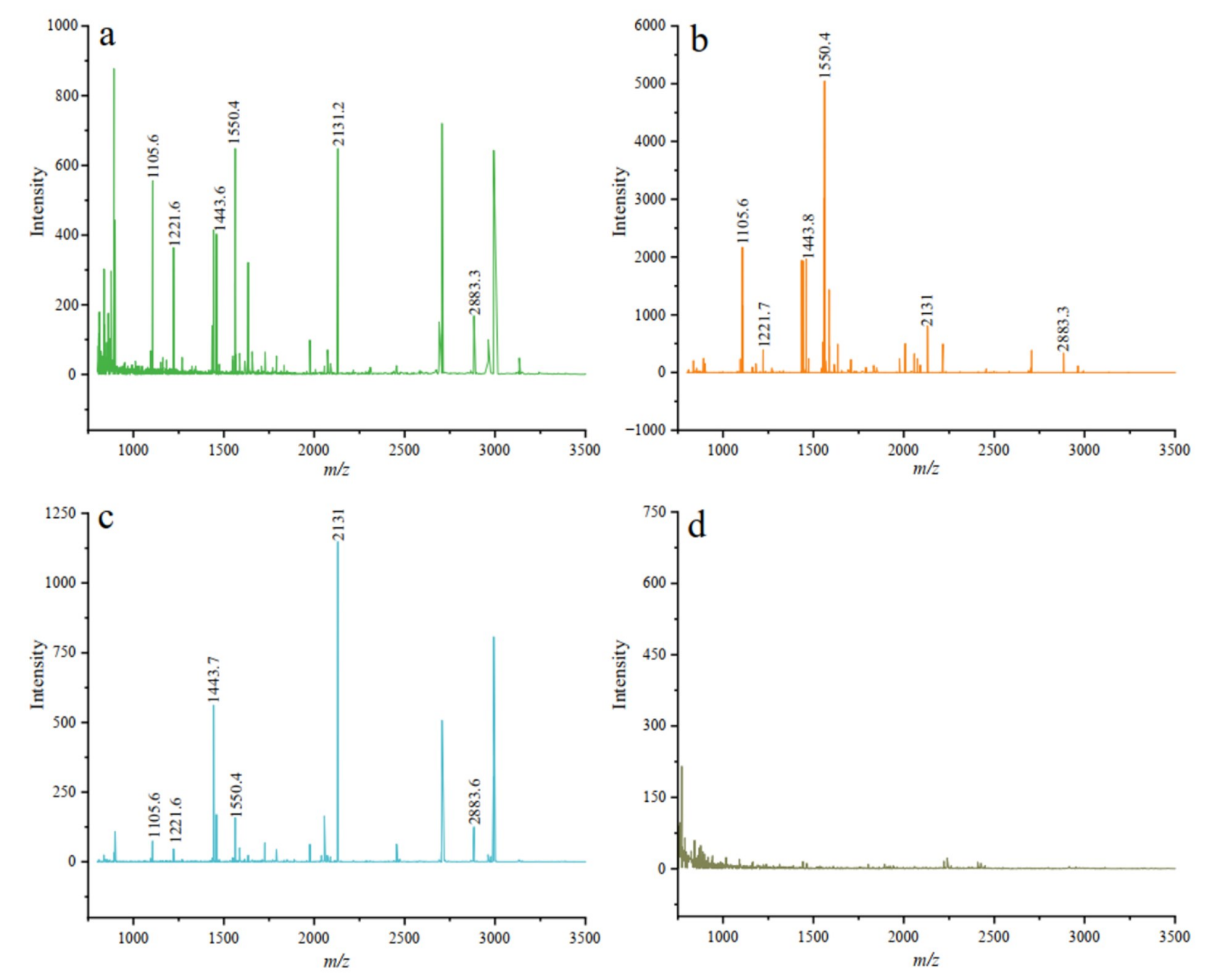
测试样本的肽质量指纹图谱

### 2.2 数据处理及数据库的比对

获得肽质量指纹图谱后进行数据处理和比对，首先使用mMass软件处理低质量信号，再提取谱图中的特征峰，并与胶原蛋白数据库进行比对^［19］^。经ZooMS实验谱图分析，3个骆驼样本的胶原蛋白保存情况较好，经比对发现，在3个样本中均检测到属于双峰驼的特征峰，证明3个样本均为双峰驼，与先前蛋白质组学的鉴定结果^［6］^一致，说明该ZooMS实验方案在晚更新世化石样本的鉴定中具有可行性。

### 2.3 ZooMS应用于实际样品检测

将该ZooMS策略应用于晚更新世古生物遗存的种属鉴定研究。共测试了40个碎片样本，其中，鉴定到种属的样本有36个（检测到3条或3条以上特征性肽段），具体种属鉴定结果见表1，鉴定成功率为90%。对比吉林大学地质学研究人员陈军和李晓波基于形态学特征给出的初步种属鉴定结果，发现仅有的13个可进行形态学鉴定的样品中，只有5个样本的鉴定结果与ZooMS一致，表明对于化石碎片，传统形态学鉴定存在一定的局限性。ZooMS鉴定中，4个样本无法获得有效谱图，判断是由于样本保存原因或者取样部位选择不当，提取的胶原蛋白含量低于MALDI-TOF MS的检出限。

**表 1:** 晚更新世古生物遗存的ZooMS鉴定结果（40个样本）

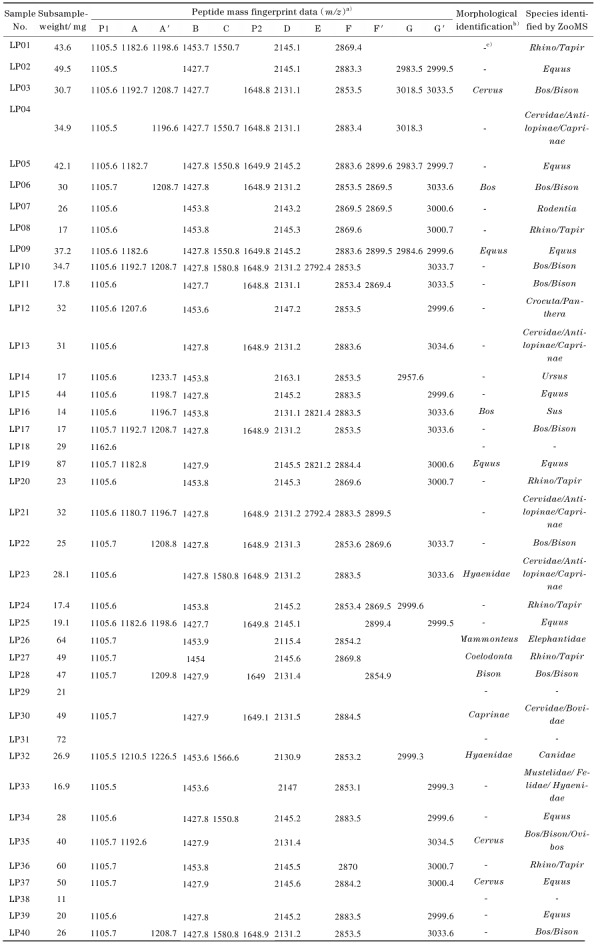

a） ZooMS peptide markers： A=COL1α2 978-990， B=COL1α2 484-498， C=COL1α2 502-519， D=COL1α2 793-816， E=COL1α2 454-483， F=COL1α1 586-618， G=COL1α2 757-789， P1=COL1α1 508-519， P2=COL1α2 292-309. Three diagnostic markers （A， F， G） exhibit dual *m/z* peaks （denoted A′/F′/G′）， indicating post-translational modifications. b） The results of morphological identification were provided by Chen Jun and Li Xiaobo from the College of Earth Sciences， Jilin University. c） Samples that cannot be identified at the species level.

### 2.4 构建古生物样本检测的标准化污染防控策略

为了减少实验中可能存在的外源蛋白质污染，并控制样本间交叉污染（第3类污染），吉林大学分子考古实验室注重提高实验人员的安全意识、应急能力、防污染意识及防控污染技术^［20，21］^。污染控制的讲解和指导贯穿于全部实验过程，实验人员全部操作在超净台中进行，并保证所有实验严格遵守无菌操作方法。实验设计多组阴性对照组，并进行多次重复实验，对仪器进行标准校正，避免因操作失误或样品污染等造成实验误差^［6］^。

## 3 教学反思及实验成果拓展应用

大学生创新训练项目模式为教学改革提供了新的思路。教师为学生提供科研热点备选方案，学生利用数据库资源查阅资料，撰写项目申报书，确定实验方案，主导实验过程，探讨使用与改进ZooMS方法，并解决实际考古问题，取得了较好的实验效果。但在实验过程中，还存在因蛋白含量过低而无法达到检出限及偶尔出现外来蛋白污染的问题，期望在后续的研究中逐步得到解决。

依托ZooMS的优化，后续以“古蛋白质组学用于中国北方古代个体食谱分析”为题开设了“大学生创新创业研究计划项目”1项，该项目将古蛋白质分析方法应用于我国北方古代人群牙结石的鉴定，获得古人直接食用动物奶制品的关键证据，为古代人群的饮食结构研究提供了新的视角。且该项目获批2023年国家级“吉林大学大学生创新训练项目”及优秀结题项目。

## 4 结语

本创新训练实验项目依托吉林大学分子考古实验室，利用公共仪器平台的优质资源，让本科学生主导展开实验项目。在教学过程中，教师遵循“以教师为辅、学生为主”的指导原则，注重与学生的密切交流，引导学生利用基质辅助激光解吸电离飞行时间质谱，深入探索基于胶原肽质量指纹图谱的ZooMS技术方法，鼓励学生们在实验过程中主动思考，积极探索，独立解决问题。通过团队协作、反复实验，建立了一套成熟的、稳定的、标准化的古生物遗存碎片的种属鉴定流程，并成功地应用于晚更新世古生物遗存碎片的种属鉴定研究。作为传统形态学及常规生物种属鉴定方法的重要补充，ZooMS技术将在古生物遗存碎片的种属鉴定研究中发挥重要的价值。利用学科间的融合交叉，从热点科学研究需求出发，通过创新训练实验项目的开展，学生的学科知识、创新思维、动手能力和科学素养都得到了显著提升，对于科学研究、人才培养和社会服务都具有重要意义。
